# Evidence-based service delivery interventions for testing, linking, treating, and retaining children and adolescents living with HIV in primary health care settings: Protocol for a systematic review

**DOI:** 10.1371/journal.pone.0269063

**Published:** 2022-06-16

**Authors:** Nande Putta, Caitlin E. Hansen, Melissa C. Funaro, Melissa Campbell, Shaffiq Essajee, Dorothy Mbori-Ngacha, Shadrack Frimpong, Shi-Yi Wang, Elijah Paintsil

**Affiliations:** 1 United Nations Children’s Fund (UNICEF), Health Programme, New York, New York, United States of America; 2 Department of Pediatrics, Yale School of Medicine, New Haven, Connecticut, United States of America; 3 Harvey Cushing/John Hay Whitney Medical Library, Yale School of Medicine, New Haven, Connecticut, United States of America; 4 Yale School of Public Health, New Haven, Connecticut, United States of America; 5 Department of Pharmacology, Yale School of Medicine, New Haven, Connecticut, United States of America; 6 Yale School of Management, New Haven, Connecticut, United States of America; Johns Hopkins School of Public Health, UNITED STATES

## Abstract

**Background:**

At the end of 2019, there were about 2.8 million children and adolescents aged 0–19 living with HIV. In contrast to pregnant women and adults, service delivery for children and adolescents living with HIV continues to lag behind with regard to access to care, components of care delivery, treatment options, and clinical and immunologic outcomes.

**Aim:**

The aim of this systematic review is to synthesize the evidence on the most effective interventions, models, programs, and strategies to optimize the delivery of services for the testing, linkage, treatment and retention of children and adolescents living with HIV globally.

**Methods:**

This review protocol is registered at PROSPERO with Registration number: CRD42020209553. The systematic review will be conducted in accordance with the Preferred Reporting Items for Systematic Review and Meta-analysis Protocols (PRISMA-P). We will use a comprehensive search strategy to search several bibliographic databases including MEDLINE, Embase, CINAHL, Cochrane Library, Global Health, and Psycinfo to identify relevant studies published in the last ten years (2010 to 2020). In addition, we will review cited and citing references of included studies. A pair of reviewers will independently screen titles, abstracts and full texts of articles, extract data from articles meeting inclusion criteria and perform quality assessments of the evidence collected. We will conduct a narrative synthesis of our findings, and if there are sufficient clinically similar studies available, we will conduct meta-analysis using a random-effects model.

**Discussion:**

This review will provide evidence on service delivery models that have been evaluated in a range of settings to efficiently and effectively locate, link, treat and retain in care, children and adolescents living with HIV. The synthesized evidence will help guide national governments and health care providers in prioritizing and adopting evidence-based service delivery approaches for children and adolescents living with HIV.

**Systematic review registration:**

PROSPERO CRD42020209553.

## Background

Nearly all HIV infections in children and adolescents occur as a result of ‘vertical’ transmission during pregnancy, at birth or during breastfeeding. The World Health Organization’s (WHO) recommendation to offer lifelong antiretroviral therapy (ART) to all pregnant women who test positive for HIV has been adopted by most WHO member countries and has been transformative in expanding access to treatment for this sub-population. In 2019, 85% of pregnant women living with HIV globally received ART and the number of new HIV infections in children was an estimated 150,000 around the world, 48% lower than the comparable estimate of 310,000 for 2010 [1]. In 2019, there were 2.8 million children and adolescents aged 0–19 years living with HIV [2]. In contrast to the progress in improving access of pregnant women to ART, in 2019, only 53% of children and adolescents living with HIV aged 0–14 years were receiving lifelong ART, and only 60% of HIV exposed infants received an HIV test within 2 months [3]. While AIDS-related mortality has fallen by 53% among children and adolescents (0–19 years) since 2000, mortality remains unacceptably high, with the majority of deaths (60%) occurring in children under five years [4].

Between 2010 and 2020, there has been unprecedented collective global efforts to reverse the HIV/AIDS narrative in children and adolescents. Notably among them were the two consecutive global frameworks focusing resources on the HIV global response for these sub-populations: 1)The Global Plan towards Elimination of New Infections among Children and Keeping their Mothers Alive, 2011–2015 [5], a Joint United Nations Programme for HIV/AIDS (UNAIDS) framework in which the 4th prong was to strengthen HIV care, treatment and support for women and children living with HIV and their families; and 2) The Start Free, Stay Free, AIDS Free Framework launched by UNAIDS in 2016, a Super-Fast-Track Framework for ending AIDS in children, adolescents, and young women by 2020 [6]. Several global initiatives unfolded within the context of these 2 frameworks including: i) the Pediatric HIV Treatment Initiative (PHTI) to spur innovation and access to improve the lives of children living with HIV [7], launched in 2014 by UNITAID to expedite development and delivery of new antiretroviral (ARV) formulations; ii) a two-year Accelerating Children’s HIV/AIDS Treatment (ACT) initiative [8], launched in 2014 by Children’s Investment Fund Foundation (CIFF) and PEPFAR focused on determining which interventions and service delivery models were most important for children and adolescents living with HIV; iii) the All-In Strategic Framework to end the AIDS epidemic among children and adolescents (aged 10–19 years) by 2030 [9] launched in 2015 by UNAIDS and UNICEF as a platform for action and collaboration to inspire a social movement to drive better results with and for adolescents through critical changes in programs and policy; and iv) the Diagnostics Access Initiative [10] launched in 2015 by a partnership including UNAIDS, the WHO, the Clinton Health Access Initiative (CHAI), PEPFAR, the Global Fund to Fight AIDS, Tuberculosis and Malaria, the US Centers for Disease Control and Prevention (CDC), the African Society for Laboratory Medicine, the United States Agency for International Development (USAID), UNITAID and UNICEF, to improve access to optimal and affordable diagnostic technologies. Despite all these efforts, there are slow and uneven improvements in recent years leading to unmet 2020 ‘superfast-track’ goals [11] in most countries [12]. This failure is a call to action and the need for critical appraisal of current service delivery methods. There is a need for more thoughtful, critical assessment of where countries are regarding reaching their treatment goals, and synthesis of evidence-base solutions.

There is broad consensus [13] among global experts and stakeholders that a combination of the right diagnostics, the right drugs, and the right service delivery approaches, will ensure greater access to treatment, improve rates of viral suppression and lower mortality for children and adolescents living with HIV. While diagnostics and drugs have clear mechanisms of development and validation, that of service delivery—which is influenced by context as well as provider and client perspectives and circumstances—relies very much on a test-and-learn iterative cycle. For service delivery to be comprehensive and consequential, it must be effective for all aspects of HIV care continuum—testing, linkage to care, treatment, and retention in care. There is a paucity of data on the effectiveness of service delivery models used in in the care of children and adolescents living with HIV. The poor antiretroviral treatment outcomes in this population have been implicated on the lack of effective interventions. Only 62% of 12- to 24-year-olds achieved 95% or greater adherence on ART [14]. In a 2015 systematic review by MacPherson and colleagues, they identified limited evidence on the effectiveness of service delivery interventions to support linkage from HIV diagnosis to ART initiation and retention in care for adolescents living with HIV [15]. Interestingly, there have been several proven interventions to improve linkage, retention, and adherence to ART in adults living with HIV that have been introduced to routine clinical care [16–18]. UNICEF has been instrumental in recognizing, designing and implementing evidence-based delivery models for prevention of mother-to-child transmission of HIV (PMTCT) and care of children and adolescents living with HIV, showcased most recently by leading the development of the service delivery framework: “Improving HIV service delivery for infants, children and adolescents: A framework for country programming” [13], in collaboration with global and national partners.

As children and adolescents continue to lag behind in the HIV response, experiencing the lowest access to services and the poorest outcomes in comparison to pregnant women and adults [4, 19]; synthesizing what has been shown to work in service delivery for these sub-populations is a critical step to providing the most useful guidance to aid national governments and health care providers in prioritizing focus.

The objective of this systematic review is to identify the most effective service delivery interventions across the HIV care continuum for children and adolescents ages 0–19 years. The specific research questions to be addressed by this systematic review is: What interventions are effective at improving HIV service delivery for children and adolescents along the HIV care continuum—testing, linkage, treatment, and retention—in primary health care including community-level settings?

## Methods

This review protocol is registered at PROSPERO with Registration number: CRD42020209553 [20]. This systematic review will be conducted in accordance with the Preferred Reporting Items for Systematic Review and Meta-analysis Protocols (PRISMA-P) [21].

### Criteria for considering studies for this review

The eligibility criteria for selecting studies for this review are as follows:

#### Types of studies

Studies that examine HIV service delivery models for children and adolescents 0–19 years of age will be included in this review. This is a quantitative review that will include published randomized trials and cluster randomized trials as well as non-randomized studies including cohort studies, case-control studies, cross-sectional studies, interrupted time series, and pre/post design studies that are age-disaggregated. We will exclude qualitative studies, reviews, case studies, protocols and clinicaltrials.gov registrations, editorials and commentaries, as well as programmatic reports, book chapters, theses, and conference abstracts/proceedings.

#### Types of participants

Studies that include children and adolescents 0–19 years of age as participants will be included in this review. Because many studies of adolescent HIV also include young adults up to age 24 years, we will also consider including these studies so as not to unnecessarily exclude important data on adolescent populations. Similarly, we will include PMTCT studies that include or impact infants or pregnant adolescents. Moreover, if a study includes both children and adults, it will be included if data is age-disaggregated, and outcomes are reported for children separately.

#### Types of interventions

An intervention refers to any service delivery models, best practice, approach, or innovations that are aimed at improving access to testing, linkage, treatment and retention for children and adolescents living with HIV. Specifically, we will consider the ‘what to do’ approaches and innovations that have been implemented in pilot form or at scale in a range of settings, to efficiently and effectively locate and test, link, treat and retain children and adolescents living with HIV, including by recognizing important facilitators and addressing common bottlenecks and barriers. This will exclude pharmacological and laboratory sensitivity and specificity studies that have been rigorously reviewed by the WHO and whose findings are represented in the WHO clinical guidelines [22, 23]. We will categorize interventions using the following locate/test-link-treat-retain continuum of HIV care approach from the UNICEF Pediatric HIV Service Delivery Framework [13]:

- Interventions to LOCATE previously undiagnosed children and adolescents, such as index family-based testing, testing in school programmes and testing of sick children.
- Interventions to LINK HIV-positive children and adolescents to treatment services, such as peer navigators and electronic registers.
- Interventions to improve access to and quality of TREATMENT for children and adolescents, such as service decentralization, measures of treatment response (e.g., viral suppression), integrated mental health counseling and disclosure support.
- Interventions to RETAIN children and adolescents in care and ensure adherence, such as peer support, appointment diaries and Short Message Service (SMS) reminders.

Combination or packages of interventions will also be considered where the effect of each distinct intervention has been measured. Similarly, packages that combine HIV and non-HIV interventions will be considered where the effect of distinct HIV interventions has been measured.

#### Comparator(s)/control

We will include studies with any comparison group (other intervention(s), usual care/delivery system, or to baseline).

#### Setting

There will be no restrictions by type of study setting.

#### Language

There will be no restrictions by language of publication and non-English articles will be translated using google translate [24].

#### Types of outcome measure(s)

The primary outcomes of interest are quantitative measures of the impact of HIV service delivery interventions on any of the HIV care continuum stages, as defined by study authors. Types of outcomes may include the following:

- Testing: access to testing (i.e., coverage), proportion accepting testing, proportion of patients tested, diagnostic yield or positivity rate (proportion of tests that return positive), turn-around time for return of results, proportion (of infants) tested receiving results, knowledge of status
- Linkage to care: proportion of patients linked to HIV care, proportion of patients enrolled into care, successful vs unsuccessful transfer or transition
- Treatment: proportion of patients initiated on ART, timing of and/or time-to-ART initiation, medication adherence (proxied also as number, rate of pill refills or pill count at review visits, medication possession ratio (MPR)), proportion who attain viral suppression, proportion with virologic failure, change in viral load and CD4 count, drug resistance
- Retention in care (proportion of individuals retained in care at 6, 12, 24, 36 months etc., according to the duration provided by study authors), lost-to-follow-up rate, attrition, engagement in care and missed visits or appointments,

Other outcomes to be included will be measures of morbidity and mortality:

- Morbidity, as reported in the included studies (e.g., AIDS- and non-AIDS defining illnesses requiring outpatient and/or inpatient management)
- Mortality (HIV-specific, all-cause), Survival

Given the multi-dimensional nature of these terms, we will not seek to define or measure access or quality as outcomes in and of themselves and will focus the analysis on direct outcomes specific to testing, linkage, treatment, and retention, as reflected above.

### Search methods for identification of studies

To identify relevant studies the authors will conduct a systematic search (S1 Table) of the following electronic databases: MEDLINE (Ovid), Embase (Ovid), Psycinfo (Ovid), Global Health (Ovid), CINAHL Complete (EBSCOhost), and the Cochrane Library. In addition to searching electronic databases, we will search for unpublished literature through targeted searches of relevant websites (S1 File). We will also hand-search the reference lists of included studies to identify additional potentially relevant articles. To capture recently published articles, a second database search will be rerun before publishing the paper. No language limit will be imposed. We will restrict the search to 2010–2020 to capture the full extent in advances and innovations in diagnosis and treatment for children and adolescents during this decade.

An experienced medical librarian (MCF) will be consulted on methodology. A medical subject heading (MeSH) analysis of known key articles provided by the research team [mesh.med.yale.edu] will be done and scoping searches will be done in each database. An iterative process will be used to translate and refine the searches. To maximize sensitivity, the formal search will use controlled vocabulary terms and synonymous free-text words to capture the concepts of HIV interventions for children and adolescents. The search strategy will be peer reviewed by a second librarian, not otherwise associated with the project, using the PRESS standard [25].

## Data collection and analysis

### Selection of studies

Search results will be pooled in the reference management software program EndNote [26] [www.endnote.com] and duplicates will be removed. If multiple reports originating from the same study are identified, these will be collated. De-duplicated study records will be imported into Covidence systematic review software [27] [www.covidence.org] for the screening and data extraction process.

Two review authors will independently screen titles and abstracts to remove clearly irrelevant citations. If the study appears to be eligible, or if the abstract does not provide enough information to determine potential eligibility, then the full text of the article will be retrieved and assessed for eligibility. Full text articles will be reviewed by two review authors independently to verify that the article meets inclusion criteria. Articles that do not meet inclusion criteria will be excluded. If there is disagreement on an article’s eligibility, this will be resolved through discussion, with the involvement of a third reviewer if necessary. The reasons for excluding full-text articles will be documented.

### Data extraction and management

Data will be extracted on all studies that meet inclusion criteria. Two review authors will conduct data extraction independently using a piloted, standardized data extraction form in Covidence, with any discrepancies resolved through discussion, and the involvement of a third reviewer if needed. For outcome measures, we will extract adjusted effect estimates and standard errors where possible.

Data extraction forms will include the following:

- Study identifier: author, year of publication
- Study design
- Country/location of the study
- Study setting and time period
- Study population characteristics: inclusion/exclusion criteria, ages of participants
- Sample size
- Description of HIV service delivery intervention, strategy, or program: main intervention components, level of HIV care continuum targeted (e.g.test-link-treat-retain)
- comparator characteristics
- Results: primary outcomes and other outcomes reported

#### Assessment of risk of bias in included studies

Two review authors will independently assess the risk of bias of included studies using the Cochrane Collaboration’s risk of bias tool for randomized studies [28], and the Newcastle-Ottawa scale for non-randomized studies [29]. Any discrepancies in the assessment of bias that arise between authors will be resolved through discussion, with the involvement of a third review author if needed.

#### Measures of treatment effect

For dichotomous outcomes, we will report the result of each study as a risk ratio with corresponding 95% confidence intervals. For continuous outcomes, we will use the mean difference between treatment arms with corresponding 95% confidence intervals. We will also report standardized mean difference.

#### Unit of analysis issues

The individual will be the primary unit of analysis. Studies will be grouped and analyzed by step along the continuum that the intervention focusses on—locating and testing, linkage, treatment or retention. For each included randomized study, two review authors will consider whether groups of individuals were randomized on an individual basis or as a group to the same intervention (cluster-randomized). Any differences will be resolved through discussion, with the involvement of a third review author if needed. We will assess whether cluster randomized control trials have appropriately adjusted for the effects of clustering in the analysis, and if this adjustment has not been performed, we will plan to correct each trial by its intraclass correlation coefficient (ICC) [28]. If this information is unavailable, we will search for relevant ICCs in similar studies and plan to correct for clustering effects if possible.

#### Assessment of heterogeneity

Statistical heterogeneity will be assessed with the I^2^ statistic, using RevMan 5.4 software [30]. Heterogeneity will be assessed as low if I^2^ is between 25–49%, moderate if between 50–74%, and high if greater than 75%; while an I^2^ less than 25% will be interpreted as no heterogeneity [31].

#### Assessment of reporting biases

When appropriate, we will assess the risk of publication bias through use of a funnel plot. The funnel plot will be assessed visually for asymmetry.

### Data synthesis

Data from each individual study included in the review will be summarized and presented in a ’Characteristics of included studies’ table. We plan to categorize each included study by the primary stage of the HIV care continuum targeted by the intervention strategy and will provide a narrative synthesis of the findings.

In terms of quantitative synthesis, we anticipate substantial variation in terms of types of pediatric HIV service delivery interventions, study settings and designs, choice of comparator, study population, and reported outcome, which may preclude meta-analysis. However, if there are sufficient clinically similar studies available (in terms of design, participants, interventions, and outcomes, where I^2^ <50%), then we will pool data from studies with similar interventions in a meta-analysis using a random-effects model in RevMan 5.4 [30], and display the results of included studies in forest plots. If we encounter substantial variability between studies, then we will not pool the results but instead will summarize the findings narratively as described above and display the results of included studies in forest plots with the summary estimate suppressed.

All analyses and reporting of the review will follow PRISMA guidelines [32]. Any amendments to the protocol will be updated in the PROSPERO registration [20].

#### Subgroup analysis and investigation of heterogeneity

If data permit, we will analyze for subgroup effects by:

- Age group (0–4 years, 5–9 years, 10–19 years)
- Gender, ethnicity/race, and country of the study
- Level of HIV care continuum targeted by the intervention (test, link, treat, retain)
- Typology classification based on the Service Delivery Framework [13] that describes nine typology types, listed as types A through I as follows:
  - Generalized higher prevalence with high MTCT rate
  - Generalized higher prevalence with high incidence among adolescents
  - Generalized higher prevalence with high MTCT rate
    **and** high incidence among adolescents
  - Generalized lower prevalence with high MTCT rate
  - Generalized lower prevalence with high incidence among adolescents
  - Generalized lower prevalence with high MTCT rate
    **and** high incidence among adolescents
  - Concentrated with high MTCT rate
  - Concentrated with high incidence among adolescents
  - Concentrated with high MTCT rate **and** high incidence among adolescents

#### Sensitivity analysis

If sufficient studies are included in this review, we will conduct a sensitivity analysis by study quality, by excluding studies with high risk of bias.

#### Confidence in cumulative estimate

Two reviewers will assess the certainty of the evidence for primary outcomes (testing, linkage, treatment, retention) using the GRADE approach [33]. The quality of evidence will be assessed across four domains: risk of bias, consistency, directness, and precision.

## Discussion

All aspects of HIV care in children and adolescents continue to lag that of HIV care in adults. Mother-to-child transmission of HIV continues to fuel the HIV epidemic in children and adolescents, particularly in resource-limited settings. About one-third of infants who are infected with HIV will survive into adolescence even without treatment [34]. At the end of 2019, there were 2.8 million children and adolescents aged 0–19 years living with HIV [2]. In contrast to the progress in improving access of pregnant women to ART, in 2019, only 53% of children and adolescents living with HIV aged 0–14 years were receiving lifelong ART, and only 60% of HIV exposed infants received an HIV test within 2 months [3]. Among the persistent challenges contributing to the lagging ART coverage in this population is weak service delivery systems with several missed opportunities for identifying infants. While AIDS-related mortality has fallen by 53% among children and adolescents (0–19 years) since 2000, mortality remains unacceptably high, with most deaths (60%) occurring in children under five years [4]. Despite a decade of unprecedented efforts to improve services and outcomes for children and adolescents living with HIV, we are failing in our global ambition to eliminate AIDS. There is a need for critical appraisal of current service delivery methods to address these gaps in HIV care. Continued failure to make substantial progress will seriously jeopardize the overall goal of ending AIDS by 2030.

There are several points along the HIV care continuum that can be targeted with effective interventions to improve care—services for locating, linking, treating, and retaining children and adolescents living with HIV. Although there is paucity of data on these interventions in HIV care of children and adolescents, they have been validated in studies in adult HIV care. Kredo et al. in a systematic review assessed three models of care: (1) ART is initiated at the hospital and maintained at the health center (partial decentralization); (2) ART is initiated and maintained at the health center (full decentralization); and (3) community volunteers with basic training delivered ART to participants at the homes [35]. They did not find any significant differences in attrition in these three models of care; they concluded that streamlining services to minimize patient facility visits, providing adequate counselling, medical and peer support, and providing incentives may decrease attrition between HIV testing and ART initiation [35]. They subsequently evaluated the quality of initiation and maintenance of HIV/AIDS care among adults in models that task shift care from doctors to non-doctors [36]. Shifting responsibility from doctors to adequately trained and supported nurses or community health workers for managing HIV patients did not decrease quality of care, and in the case of nurse-initiated care, there were fewer patients lost to follow-up [36]. In a randomized clinical trial investigating whether offering optional home initiation of HIV care after HIV self-testing might increase demand for ART initiation compared with HIV self-testing accompanied by facility-based services only, MacPherson et al. found that among Malawian adults offered HIV self-testing, optional home initiation of care compared with standard HIV care resulted in a significant increase in the proportion of adults initiating ART [37].

There are few studies on HIV-infected adolescents or children and the HIV care continuum and have found their outcomes to be poor and worse than adults. Rates of retention in care prior to ART initiation, retention in care after ART initiation, and adherence to treatment are substantially worse for adolescents than adults [38, 39]. In a recent systematic review, MacPherson et al. identified limited evidence on the effectiveness of interventions to support adolescents’ linkage from HIV diagnosis to ART initiation, retention on ART and adherence to ART based on small numbers and quality of the studies [15]. To our knowledge, this systematic review will be the most comprehensive review of service delivery models for children and adolescent HIV care that has been conducted to date. Findings will provide a comprehensive summary of the evidence for the effectiveness of interventions to improve a broad range of outcomes related to pediatric HIV service delivery. In addition to publishing the review upon completion, the results will be shared with global partners, national governments, implementing partners and frontline service providers to inform policy, planning, service delivery decision making, and strategies to achieve the UNAIDS 95-95-95 global HIV target in children and adolescents. The UNICEF Pediatric Service Delivery Framework [13] will also be updated to reflect the findings of the systematic review.

## Supporting information

S1 Checklist Completed PRISMA checklist. (DOCX)

S1 Table Search strategy Ovid MEDLINE search strategy. (DOCX)

S1 File Grey/unpublished literature search list. (DOCX)
