# Supplementary material for: Evidence-based service delivery interventions for testing, linking, treating, and retaining children and adolescents living with HIV in primary health care settings: Protocol for a systematic review
Source: PLoS One. 2022 Jun 16;17(6):e0269063. doi: 10.1371/journal.pone.0269063 (PMC9202933; doi:10.1371/journal.pone.0269063)
Supplement: S1 Table — (DOCX) [file pone.0269063.s002.docx]

## **S1 Table. Search strategy Ovid MEDLINE Search Strategy**

| search line | search strategy | # results |
| --- | --- | --- |
| 1 | exp pediatrics/ or exp child/ or exp adolescent/ or exp infant/ or exp young adult/ or (adolescent or adolescents or adolescence or babies or baby or child or children or infancy or infant or infants or juvenile or juveniles or kid or kids or newborn or new-born or newborns or new-borns or paediatric or paediatrics or pediatric or pediatrics or perinatal or pubescence or pubescent or teen or teenager or teenagers or teens or toddler* or young adult* or young people or young person* or youngster* or youth*).tw,kw. | 4573354 |
| 2 | exp HIV Infections/ or exp HIV/ or (hiv or human immunodeficiency virus or human immunedeficiency virus or human immune deficiency virus or human immune deficiency virus or acquired immunodeficiency syndrome or acquired immune deficiency syndrome or acquired immuno deficiency syndrome or acquired immune deficiency syndrome or (human immun* adj2 deficiency virus) or (acquired immun* adj2 deficiency syndrome)).tw,kw. | 317381 |
| 3 | exp Community Health Workers/ or (((Care guide* or Clinic* or Coach* or Community based or Community health or Community level or Community service* or Community wide or (Decentralized adj3 (healthcare or care)) or delivery) adj2 health service*) or Delivery service intervention* or Facilities or Family advocate* family based care or family centered care or Front line or Front line service* or Health advisor* or health advocate* or Health center* or Health coach* or Health educator* or Health facility* or Health interpreter* or Home based or Home care or Lay health advisor* or Lay health worker* or Liaison* or Outreach worker* or Patient navigator* or Peer counselor* or Primary health care or Primary health service* or Primary service* or Primary care or Promotora* or referral hospital* or residential facility* or Self health care or vertical programm* or vertical program*).tw,kw. | 407353 |
| 4 | 1 and 2 and 3 | 5516 |
