# Supplementary material for: Evidence-based service delivery interventions for testing, linking, treating, and retaining children and adolescents living with HIV in primary health care settings: Protocol for a systematic review
Source: PLoS One. 2022 Jun 16;17(6):e0269063. doi: 10.1371/journal.pone.0269063 (PMC9202933; doi:10.1371/journal.pone.0269063)
Supplement: S1 File — (DOCX) [file pone.0269063.s003.docx]

## **S1 File. Grey/unpublished literature search list**

- [www.unicef.org](http://www.unicef.org)
- <https://www.state.gov/pepfar/>
- <https://www.usaid.gov/>
- <https://www.ampathkenya.org/>
- <https://www.cdc.gov>
- <https://www.pedaids.org>
- icap.coulmbia.edu
- cmmb.org
- unaids.org
- who.int
- crs.org
- [www.africaid-zvandiri.org](https://nam05.safelinks.protection.outlook.com/?url=http%3A%2F%2Fwww.africaid-zvandiri.org%2F&data=02%7C01%7Ccaitlin.hansen%40yale.edu%7Cae41908c151c4280f8f808d85aa33edc%7Cdd8cbebb21394df8b4114e3e87abeb5c%7C0%7C0%7C637359004510375998&sdata=q8OaisID%2FRty3cdpzzhnvANQQky9cWHUJvj9vuRPzBo%3D&reserved=0)
- oikoumene.org
- teampata.org
- viivhealthcare.com
- [www.fhi360.org](https://nam05.safelinks.protection.outlook.com/?url=http%3A%2F%2Fwww.fhi360.org%2F&data=02%7C01%7Ccaitlin.hansen%40yale.edu%7Cae41908c151c4280f8f808d85aa33edc%7Cdd8cbebb21394df8b4114e3e87abeb5c%7C0%7C0%7C637359004510375998&sdata=XWb7nlSzh7f1SHN5bI8MnENa5W8EPe4HbpcfrVcF3NA%3D&reserved=0)
- [www.elmaphilanthropies.org](https://nam05.safelinks.protection.outlook.com/?url=http%3A%2F%2Fwww.elmaphilanthropies.org%2F&data=02%7C01%7Ccaitlin.hansen%40yale.edu%7Cae41908c151c4280f8f808d85aa33edc%7Cdd8cbebb21394df8b4114e3e87abeb5c%7C0%7C0%7C637359004510386001&sdata=M57pOGfYyA5AQY64uKvszgmOcoiWZZiZUlqSm5Y9Iaw%3D&reserved=0)
- aidsfonds.org
- clintonhealthaccess.org
- anecca.org
- childrenandhiv.org
- childrenandaids.org
- pactworld.org
- iasociety.org
- bcm.edu
- tingathe.org
